# Customising global climate science for national adaptation: A case study of climate projections in UNFCCC’s National Communications

**DOI:** 10.1016/j.envsci.2019.07.015

**Published:** 2019-11

**Authors:** Maurice Skelton, James J. Porter, Suraje Dessai, David N. Bresch, Reto Knutti

**Affiliations:** a Institute for Environmental Decisions, Department of Environmental Systems Science, ETH Zürich, Switzerland; b Federal Office of Meteorology and Climatology MeteoSwiss, Operation Center 1, P.O. Box 257, 8058, Zurich Airport, Switzerland; c Department of Geography, King’s College London, London, WC2R 2LS, UK; d Sustainability Research Institute and ESRC Centre for Climate Change Economics and Policy, School of Earth and Environment, University of Leeds, LS2 9JT, UK; e Institute for Atmospheric and Climate Science, Department of Environmental Systems Science, ETH Zürich, Switzerland

**Keywords:** Climate projections, climate scenarios, climate information, adaptation, geographical imbalance, customisation of climate science

## Abstract

Countries differ markedly in their production of climate science. While richer nations are often home to a variety of climate models, data infrastructures and climate experts, poorer sovereigns often lack these attributes. However, less is known about countries’ capacity to use global climate science and customise it into products informing national adaptation. We use a unique global dataset, the UNFCCC National Communications, to perform a global documentary analysis of scientific submissions from individual countries (n = 189). Comparing countries’ climate projections with their competence in publishing climate science, our research examines the existence of geographical divides. Although countries proficient in publishing climate science use more complex climate modelling techniques, key characteristics of climate projections are highly similar around the globe, including multi-model ensembles of Global Circulation Models (GCMs). This surprising result is made possible because of the use of pre-configured climate modelling software packages. One concern is that these tools restrict customisation, such as country-specific observations, modelling information, and visualisation. Such tools may therefore hide a new geographical divide where countries with higher scientific capacities are able to inform what goes into these software packages, whereas lower scientific capacity countries are dependent upon these choices – whether beneficial for them or not. Our research suggests that free-to-use modelling and training efforts may unwittingly restrict, rather than foster, countries’ capacity to customise global climate science into nationally relevant and legitimate climate information.

## Highlights

• Most countries strongly committed to explore climate futures with climate models.
• Countries proficient in climate science have most sophisticated climate projections.
• Plug-and-play software producing projections create new national dependencies.
• Ability to customise global climate science to a country limited to the proficient.
• Efforts increasing data availability mask gaps in national capacity to customise it.

## 1 Introduction

If countries are to adapt to the impacts of climate change, it’s critical they possess the scientific capacity needed to produce knowledge on a relevant scale and to translate it into policies to inform local decision-making (Ho-Lem et al., 2011). Such thinking sits at the heart of the Intergovernmental Panel on Climate Change (IPCC) efforts to synthesise climate science to inform policies under the United Nations Framework Convention on Climate Change (UNFCCC). Yet not all countries are able to produce scientific climate information, or to the same extent. Studies have shown that rich, high emissions countries publish the bulk of climate research (e.g. Haunschild et al., 2016; Pasgaard and Strange, 2013). This ‘geographical imbalance’ (Pasgaard et al., 2015) makes it challenging to inform policies equally if peer-reviewed publications are favoured. As a result, ‘who’ produces climate science, and even ‘where’ climate science is produced, can have far reaching effects for the commitment to UNFCCC agreements (Corbera et al., 2015; Hulme and Mahony, 2010) as well as the commitment to local adaptation efforts too (Blicharska et al., 2017; Miguel, 2017; Lahsen, 2007).

To understand these geographies of climate science, it’s crucial to examine why differences in the publication of climate science have emerged and what differences exist over countries’ capacity to customise global climate science. A major challenge here is how to compare countries with different characteristics (e.g. size, wealth, education, stability). Measuring scientific outputs by peer-reviewed publications has proved a reliable method for highlighting the volume and geographic distribution of climate research (Haunschild et al., 2016; Pasgaard et al., 2015; Karlsson et al., 2007). But interpreting such metrics as capacity to customise climate science entails the assumptions that all countries have similar interests in publishing climate science, as well as similar capacities to contribute research (cf. Dike et al., 2018). An alternative approach is to compare the ability of countries when using and producing (adaptation-relevant) climate science where the objective is the same for all involved. Any deviation from common reporting requirements – either going above and beyond or failing to meet set standards – would provide an indication of different scientific capacities between countries, including those with few peer-reviewed publications.

To do this, our paper presents a global comparison of climate projections’ characteristics reported in UNFCCC National Communications, as a proxy of a country’s capacity to produce nationally relevant, adaptation-focused scientific climate information. Section 2 summarises the literature on the geographies of climate science. Section 3 explains how we collected the data and how we classify countries according to their competence in publishing climate science. Section 4 explores differences in reporting climate projections and countries’ compliance with UNFCCC requirements. Section 5 identifies similarities and variances in the modelling characteristics, and contrasts these with countries’ publication competence. Section 6 details how the reported climate futures^1^ are similar, regardless of countries’ quantity and quality of publications. Sections 7 and 8 offer a discussion about the emergence of a new, and mostly hidden, geographical imbalance in the way countries are supported to customise climate science for national decision-making.

## 2 On the geographies of climate science

Bibliometric studies have repeatedly revealed a geographical imbalance over the distribution of peer-reviewed climate publications (Haunschild et al., 2016; Pasgaard and Strange, 2013; Karlsson et al., 2007); and the authorship of IPCC reports (Corbera et al., 2015; Ho-Lem et al., 2011). Nearly half of all non-Annex 1 countries (45%) had no authors contributing to IPCC’s first Assessment Reports between 1990 and 2007 (FAR, SAR, TAR, AR4) (Ho-Lem et al., 2011). Furthermore, a positive correlation exists between the wealth, education attainment, number of climate publications and the number of IPCC authors of a country (Ho-Lem et al., 2011; Karlsson et al., 2007) as well as the stability of institutional arrangements within it (Pasgaard et al., 2015; Pasgaard and Strange, 2013). In turn, historically the largest carbon emitters, and arguably the less vulnerable to climate change, have focused their research towards mitigation, not adaptation which most interests highly vulnerable countries (Pasgaard et al., 2015).

Moreover, critical scholars argue that the ambitions of countries to produce world-leading scientific climate knowledge^2^, and climate models specifically, are shaped by varying histories and politics, and can (unwittingly) create geopolitical entanglements (Mahony and Hulme, 2018). For instance, Mahony and Hulme (2016) describe the motivations behind the establishment of the UK Met Office Hadley Centre to produce ‘sound science’ for politicians in order ‘to develop a trust[worth]y model of one’s own’ (Mahony and Hulme, 2016: 465). UK politicians thought it necessary to balance and influence the IPCC with a specialist British entity. ‘The capacity to predict was seen as allied to the capacity to adopt a political stance independent of both Europe and the US’ (ibid). To this day, the Met Office Hadley Centre continues to have a national as well as international agenda; the latter illustrated by the release of a free-to-use regional climate modelling software package known as PRECIS, designed for countries with lower scientific capacities to make climate risk assessments (Mahony and Hulme, 2012).

With such socio-political ideologies guiding climate model development, the central role of climate models in climate science and policymaking (cf. Shackley et al., 1998; Skelton et al., 2019), and the hegemony of only a few countries producing climate models, resistance to the application of climate research built elsewhere and for different purposes can be anticipated. Indeed, Myanna Lahsen (2007) has revealed how Brazilian policymakers distrusted joint climate science projects – between the global north and global south – believing them to be another way in which unequal power relations are entrenched by furthering the interests of the richer countries, not those of Brazilian scientists or politicians. Without a global climate model, emerging economies such as Brazil, can find it difficult to ‘act sovereignly’ in international climate negotiations (Miguel, 2017: 7). With ‘climate modeling appearing as a strategic science’ for emerging economies the ‘national production of this type of technoscience [climate models] is an important pragmatic geopolitical approach for countries of the South wishing to occupy positions within the international climate change framework’ (Miguel, 2017: 8).

While Brazil and China have their own climate modelling centres, other nations may lack the same level of technical infrastructure and investment. Such observations have led Blicharska et al. (2017) to urgently call for this so-called ‘north-south divide’ to be tackled. Fostering ‘Post-Paris long term climate [science] capacity’ requires moving away from the ‘fly in fly out’ climate science consultancy paradigm to a mode of training younger climate scientists also within countries’ universities (Nasir et al., 2018: 130f; Dike et al., 2018).

Such studies, and calls-for-action, all problematize the idea of a value-free science which is legitimate around the world and can be imported and exported without encountering local or political friction. Yet science is always infused with national interests, histories, and politics that makes it more or less problematic to apply in different contexts. Both the UK and Brazil case studies above reveal similar political perceptions about climate models, regardless of their competence in climate science. However, the comparison by Skelton et al. (2017) of how climate science leaders produced their national climate projections shows that leading countries also rely heavily on climate models produced outside their countries (partly to account for their structural uncertainty, cf. Parker, 2010). Swiss, Dutch and British climate scientists focused far more on customising these global homogenous datasets into nationally legitimate climate science, even to the degree of (subconsciously) tailoring it to the countries’ social and epistemic values. This tailoring included climate scientists interacting with certain users of climate scenarios in a nationally particular way (through user representation, broad participation, or elicitation), as well as scientists favouring research that reflected nationally particular assumptions of science for ‘good’ decision-making (such as, peer-reviewed, novel and innovative, or user friendly) (Skelton et al., 2017). While climate science has been a supra-national, global endeavour since its inception a hundred years ago (cf. Edwards, 2010), its global knowledge products tend to homogenise local differences and meaning instead of enriching the global datasets with local socio-political values (Hulme, 2010). As such, studies that focus only on the origin of climate science miss the political and scientific importance of customising that global climate science into something that is nationally legitimate, salient and credible (Cash et al., 2003).

## 3 Data and methods

To meaningfully compare countries’ capacity to produce nationally relevant climate science for decision support, we conducted a documentary analysis of countries’ most recent National Communication (n = 189). National Communications are a unique global dataset, which report on the progress of a UNFCCC member’s mitigation and adaptation commitments. Analysis of this dataset has been undertaken for a number of comparative studies, from the formulation and implementation of climate policies across different countries (Albrecht and Arts 2005) to tracking progress made on global adaptation by differentiating global leaders from the laggards (Lesnikowski et al., 2015). UNFCCC provides clear guidance (UNFCCC, 2008) and training sessions (UNFCCC, 2012, 2016) on what National Communications should include. These submissions are authored and officially signed-off by the countries in question. Analysing deviations from these reporting requirements – by either going beyond or below expected standards – are indicators for national differences in using and customising climate science, potentially revealing a geographical imbalance.

We downloaded the most recent National Communication submissions from the UNFCCC website, submitted to the UNFCCC between 30.10.1999 and 31.12.2016. Each one of the n = 189 submissions was weighted equally, irrespective of when it was written (see Supplementary Fig. 1). We then coded all the submissions manually. This involved reading each document and recording answers to a range of questions concerning climate projections in an Excel database. These questions included (i) were Global Circulation Models (GCMs) or Regional Climate Models (RCMs) used; (ii) what downscaling techniques were used (e.g. statistical/dynamical); (iii) how many emissions pathways were used; and (iv) which timeframes were used (e.g. > 2080s) (see Supplementary Materials for a full list).

**Fig. 1:**
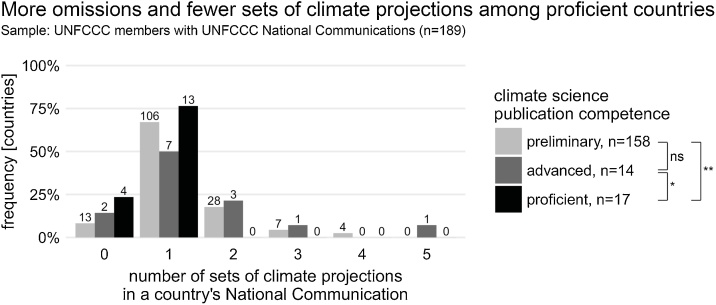
Number of sets of climate projections reported in UNFCCC National Communications, grouped by countries’ climate science publication competence. Most countries submitted a single set of climate projections. Distributions are compared with Mann-Whitney U tests comparing two country groups; ns denotes ‘not significant’, *p<.05, **p<.01.

Various countries reported multiple sets of climate projections, for example one covering the entire national territory and a second one only for a particular administrative region. To distinguish these, we define a ‘set of climate projections’ as one product, potentially encompassing multiple climate models, outputs, and emissions pathways to describe multiple yet coherent climate futures. This includes, for instance, aggregating climate information from multiple climate models and/or climate model runs for one emissions pathway. When a country reported more than one set of climate projections, we applied two criteria to narrow the selection down to one set. First, we prioritised the set of climate projections that focused on the entire country, rather than a single geographical region. Second, we selected the climate projection that contained higher concentrations of information (measured as the relative space used by text descriptions, graphs and tables).

In order to contrast countries’ capacity to produce climate projections with their general competence in publishing climate science, we classify countries according to the quantity and quality of their publications. For this, we draw on Haunschild et al. (2016) comparing more than 200,000 peer-reviewed publications and their citations between 1980 and 2015, using keywords similar to ‘climat* change’. By selecting the proportion of papers belonging to the 10% most frequently cited – the indicator PP_top10%_ – Haunschild et al. (2016) ranked countries’ competence in publishing climate change research. We use this indicator to create three levels of publication competence: proficient, advanced, and preliminary. We define proficient as a PP_top10%_>20 and >1000 published papers between 1980 and 2015. N = 17 countries fit this category, including many European countries as well as Australia, New Zealand, and the US (see Supplementary Materials for full list). All proficient countries have dedicated climate modelling centres developing GCMs and/or RCMs. Countries classified as advanced have >1000 published papers but a PP_top10%_<20. N = 14 nations meet these criteria, including Brazil, China, Greece, India, Israel, and Japan. In advanced nations, climate science is funded and publications are numerous, but they are not often excellent. The remaining n = 158 countries have a preliminary competence with <1000 published papers (which may or may not be excellent). This includes, for example, all African countries except South Africa, Chile, Croatia, Liechtenstein, Tajikistan, and Viet Nam. In preliminary countries, publishing large amounts of climate science is a challenge, either due to being a poorer, highly populated country or a smaller, richer nation.

Lastly, our research highlights, and is subject to, some limitations with the UNFCCC National Communications dataset. Amongst the countries that failed to submit any climate projections in their National Communications, further research revealed that Australia (CSIRO and Bureau of Meteorology, 2007), Canada (Barrow et al., 2004) and Spain (Gutiérrez et al., 2012), have in fact all produced national climate projections. Why these climate projections were not included in the submissions is unclear. Such observations are, however, helpful in revealing the challenges of working with global datasets where reporting requirements are either inconsistently met or simply ignored. Further, the voluntary nature of reporting National Communications for non-Annex 1 countries results in climate projections produced at irregular intervals (see Suppl. Fig. 1). Thus, n = 10 countries reported climate projections within their National Communications submitted between 1999 and 2008, complicating comparison as more recent submissions have more climate models available. However, the majority (n = 120/189, 63%) of National Communications have been submitted in the short time span between 2013 and 2016.

## 4 Did all countries include climate projections in their National Communications?

Of the 196 UNFCCC member states, 189 countries submitted National Communications^3^ . In 90% (n = 170/189) of cases, countries’ submissions included climate projections as part of their vulnerability and adaptation assessment (Fig. 1). While the UNFCCC reporting guidelines don’t prescribe how many climate projections should be reported, a broad consensus emerged. The majority of countries (n = 126/189, 67%) provided a single, national, set of climate projections, while a minority of countries (n = 43/189, 33%) chose to report multiple sets of climate projections. Multiple climate projections often focused on several different spatial or administrative scales (e.g. regions, cities and airports), and could be used to inform local government policies and decision-making.

Crucially, Fig. 1 highlights that the global distribution of climate projections, based on UNFCCC National Communications, plays out differently to what might be expected from the literature (cf. Blicharska et al., 2017; Haunschild et al., 2016; Pasgaard and Strange, 2013). Climate projections are produced and available across the globe – but more often (and in higher numbers) in countries classified as preliminary and advanced (n = 145/158, 92%) rather than proficient in publishing climate science (n = 13/17, 76%). A Kruskal-Wallis H test confirms that the number of reported climate projections is indeed (inversely) correlated to a country’s publication competence (X^2^(2, n = 189) = 8.0, p < .05). This is even more surprising given that the vulnerability section reporting guidelines for non-Annex I countries of the UNFCCC are voluntary. Aware of various countries’ capacity and resource constraints, the UNFCCC ran several ‘hands-on training workshops’ before the submission of National Communications, introducing free-to-use and well-established software tools such as PRECIS and MAGICC/SCENGEN (UNFCCC, 2016).

Among the few preliminary countries failing to report climate projections are n = 5 oil-rich countries (Angola, Bahrain, Egypt, Qatar, and the United Arab Emirates) and n = 3 Small Island Developing States (SIDS). This finding echoes that of Pasgaard et al. (2015) where SIDS are correlated with lower numbers of climate change publications. Among the proficient countries omitting climate projections in their National Communications are interestingly some which had them readily available, such as Australia (CSIRO and Bureau of Meteorology, 2007), Austria (Loibl et al., 2009) and Canada (Barrow et al., 2004)^4^ .

## 5 What climate model characteristics do National Communications submissions share?

Of the n = 170 (out of 189) National Communications that provided climate projections, our research found that while the complexity of the methods used correlates significantly with a country’s climate science publication competence, the number of climate models (e.g. Global Circulation Models (GCMs) or Regional Climate Models (RCMs)) is independent of a country’s publication record.

### 5.1 Climate modelling complexity

**Fig. 2:**
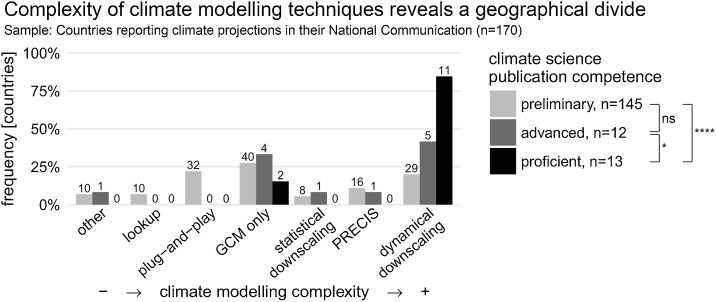
Distributions of climate modelling complexity, ranked from less complex (left) to more complex methods (right), grouped by countries’ publication competence. More complex modelling efforts allow more customisation, but require a higher level of understanding and technoscientific modelling infrastructures. Distributions are compared with Mann-Whitney U tests comparing two levels of publication competence; ns denotes ‘not significant’, *p<.05, ****p<.0001.

As shown in Fig. 2, we created a rank order from the least to the most complex climate projections approaches. For instance, while some techniques don’t require specialist knowledge to produce climate projections, others allow a high level of customisation with different sets of observations, models or statistical methods. Modelling efforts were classified according to one of seven ranks:

1 *Other.* No details provided about the methods or data sources used. N = 1 advanced and n = 10 preliminary nations fitted this category.
2 *Lookup.* Existing datasets, such as the United Nations’ Climate Change Country Profiles (McSweeney et al. 2010), are used to insert tables or figures into the National Communications. No data customisation is possible.
3 *Plug-and-play.* Software packages including MAGICC-SCENGEN (Wigley, 2008) and SimCLIM (Warrick et al., 2005) are used to calculate climate futures using a simple energy-balance model with pattern-scaling. Some data customisation is possible.
4 *GCM only.* Raw data is downloaded from portals such as ‘Climate Explorer’ (Trouet and van Oldenborgh, 2013) and projections produced using one or multiple GCMs. However, the spatial resolution of GCMs (100 km and more) cannot account for topographical features such as mountain ranges or islands.
5 *Statistical downscaling.* GCM outputs are downscaled using statistical techniques to achieve a higher spatial resolution. A high level of technical skill is required to perform downscaling competently (Wilby et al., 2002).
6 *PRECIS.* Tailored to researchers in countries with lower coverage of observational datasets, the RCM PRECIS requires solid expertise while running on a Linux-based PC with a simple user interface.
7 *Dynamical downscaling.* A highly demanding technical approach for producing high-spatial resolution outputs (e.g. < 25 km) using RCMs. Freedom for customisation is high. However, RCMs have issues with nonlinear feedbacks and miss long-distance climate linkages (teleconnections).

Fig. 2 reveals a clear-cut geographical imbalance in which countries’ capacity to use complex modelling techniques and to customise climate model output into national climate projections is highly correlated with their competence of publishing climate science (Kruskal-Wallis H test: X^2^(2, n = 170) = 19.6, p < .0001). Further Mann-Whitney U comparisons between only two competence levels (Fig. 2, legend) reveals that distributions differ most for proficient countries, which almost all mapped onto a single, the most complex, category (i.e. dynamical downscaling, n = 11/13, 85%). This contrasts with most preliminary countries (n = 82/145, 57%) choosing a less complex modelling method (i.e. lookup, plug-and-play, GCM only). The lack of scientific infrastructures and data availability may help explain the preference for less demanding climate modelling approaches. For instance, one of the advantages of plug-and-play methods, such as MAGICC-SCENGEN, is that they can be stored on USB devices and run offline, getting away from internet bandwidth problems. Furthermore, empirical studies have shown that simple energy balance models can perform surprisingly well compared to more complex climate models (e.g. GCMs) but require a fraction of the skill, time, and technoscientific infrastructure (Shackley et al., 1998).

That said, nearly a third of countries with preliminary numbers of climate science publications (n = 45/145, 31%) made use of the two most complex modelling approaches: PRECIS and dynamical downscaling, both using RCMs. In n = 16 cases, output from the UK Met Office’s freely available PRECIS model (Jones et al., 2004) was used. Another n = 16 countries with preliminary publication competence are smaller European nations benefitting from pan-European RCM modelling projects such as ENSEMBLES (van der Linden and Mitchell, 2009). These two factors help explain why preliminary and advanced nations differ *almost* significantly in their distributions (Mann-Whitney U test: X^2^(1, n = 157) = 3.6, p = .06).

A geographical imbalance also emerged when analysing where the underlying climate projections methods originated. Many of the modelling tools were developed by Anglophone scientists for explicit use outside their own countries, with focus on global applicability and user orientation. MAGICC-SCENGEN (Wigley, 2008) and the UN Climate Change Country Profiles (McSweeney et al., 2010) are from the United States; SDSM (Wilby et al., 2002) and PRECIS (Jones et al., 2004) are from the United Kingdom; and SimCLIM (Warrick et al., 2005) is a commercial product from New Zealand. The two continental European projects are different in this regard: the Dutch ClimateExplorer (Trouet and van Oldenborgh, 2013) is a database of GCM simulations (without direct means to produce climate projections), while Germany funded a science partnership with South Africa producing nationally-specific climate projections (DEA, 2013).

### 5.2 Number of climate models used

**Fig. 3:**
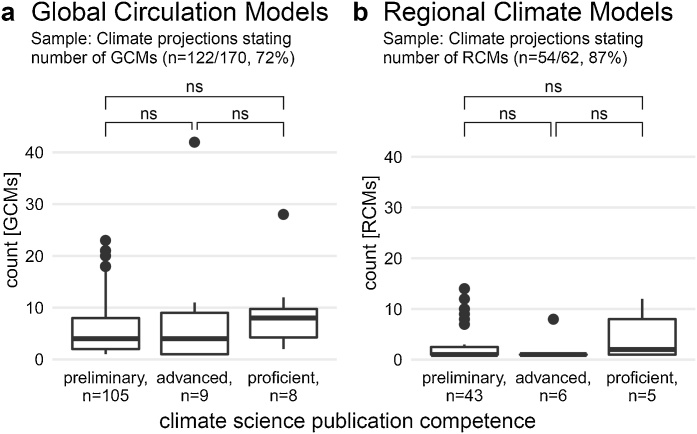
a and 3b – Distributions of the number of Global Circulation Models (GCMs, a) and Regional Climate Models (RCMs, b) used in climate projections, grouped by countries’ publication competence. While Fig. 3a shows that multi-model GCM ensembles are common independently of the country classification, most climate projections with RCMs have used only a single one (Fig. 3b). Mann—Whitney U tests performed for comparing two country groups supports their independence; ns denotes ‘not significant’. NB: Bold line denotes the median; box the 25^th^ and 75^th^ percentile; whiskers the 5^th^ and 95^th^ percentile; points are outliers.

Fig. 3a is a boxplot showing the number of Global Circulation Models (GCMs) used to produce climate projections in countries’ National Communications, grouped by their publication competence. Excluding the outliers, where Argentina used 42 GCMs and Finland 28 GCMs, the data shows little difference in the distributions of GCMs. While the median number of GCMs is higher for proficient countries, a Kruskal-Wallis H test shows that the distributions are independent of countries’ publication competence (X^2^(2, n = 122) = 2.14, p = .34). The wide use of multiple GCMs is thus encouraging, given that multi-model ensembles inform about certain aspects of structural uncertainties in climate modelling practices (Kreienkamp et al., 2012; Knutti et al., 2010; Parker, 2010). The availability of multiple GCMs in less complex climate projections may well have to do with up to 20 GCMs included in MAGICC-SCENGEN (Wigley, 2008). In addition, more recent submissions benefitted from projects such as the Coupled Model Intercomparison Project (CMIP, e.g. Meehl et al., 2014) facilitating the download of multi-model output.

Fig. 3b shows the distribution in the number of Regional Climate Models (RCMs) used by those countries employing PRECIS or dynamical downscaling (see Fig. 2). A Kruskal-Wallis H test corroborates the visual impression that the distributions of RCMs don’t differ significantly with publication competence (X^2^(2, n = 54) = 5.17, p = .08). However, Fig. 3b indicates that the recommended use of multi-model ensembles (Knutti et al., 2010) hasn’t as yet been transferred to the use of multiple RCMs as well. Only European countries, thanks to the European ENSEMBLES project (van der Linden and Mitchell, 2009), have had multiple RCM simulations available for their national territory. This may change as the global availability of RCMs increases through initiatives such as CORDEX (Giorgi et al., 2009). However, it remains to be seen how much countries with less publication experience and less technoscientific infrastructure can harness these additional sources, as computational complexities, dataset size, time required, and resources needed all increase.

## 6 What type of climate futures do countries report?

To be able to inform adaptation decisions, it’s key to not only have the ability to use multiple models, but also to incorporate different socio-economic conditions and timeframes to understand how different climate futures can develop. Too many timeframes and/or emissions pathways can result in an inability to work through different variations, creating a decision-making paralysis. Too few, by contrast, locks decision-makers into a deterministic view that discounts the importance of uncertainty (Hulme and Dessai, 2008; Parker, 2010). To that end, this section highlights: (i) how many timeframes were considered (e.g. up to 2050s or 2090s); and (ii) how many emissions pathways were used (e.g. single vs. multiple).

**Fig. 4:**
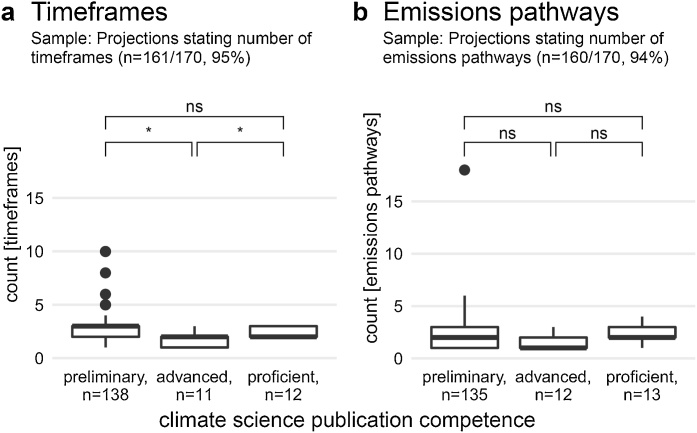
Distributions of the number of timeframes (a) and emissions pathways (b), grouped by countries’ publication competence. Significances of Mann-Whitney U tests between two levels highlight that only advanced countries used significantly fewer timeframes; ns denotes ‘not significant’, *p<.05. NB: Bold line denotes the median; box the 25^th^ and 75^th^ percentile; whiskers the 5^th^ and 95^th^ percentile; points are outliers.

First, the vast majority of countries – independently of their publication competence in climate science – used multiple timeframes (n = 129/170, 76%) up to the end of the century (Fig. 4a). IPCC guidance notes that ‘[t]he length of time period considered in the assessment studies can significantly affect results’ (Knutti et al., 2010: 11). In response, the UNFCCC recommended that countries ‘consider time frames ranging from 2030 to 2100’ in order to adequately incorporate climatic changes arising from socio-economic factors in longer-term (e.g. after the 2060s) (UNFCCC, 2008: 12; see also Hawkins and Sutton, 2009). While comparing all three country classifications simultaneously shows no significantly different distributions in the number of timeframes reported (Kruskal-Wallis H test: X^2^(2, n = 161) = 4.9, p = .08), comparing only two publication competence levels reveals that advanced countries reported significantly fewer timeframes than both proficient and preliminary countries (Fig. 4a). This has mainly to do with the third of advanced countries reporting only a single timeframe (n = 4/12, 33%).

Second, the number of emissions pathways (Fig. 4b) is independent of a country’s publication competence (Kruskal-Wallis H test: X^2^(2, n = 160) = 3.4, p = .18). Whilst UNFCCC guidance (UNFCCC, 2008: 12) acknowledges that ‘developing baseline scenarios can be complex and time-consuming’, it is recommended that at least two emissions pathways be selected – one high and one low temperature response – to capture the uncertainty around future greenhouse gas emissions (Kreienkamp et al., 2012).

Overall, n = 13 preliminary and n = 3 advanced nations reported only a single emissions pathway and a single timeframe, depicting thus a deterministic view on only a single, mostly pessimistic, climate future. These countries most often reported a mid- to long-term future with a high (e.g., A1B) (n = 8) or very high (e.g., RCP8.5) (n = 5) emissions pathway. No country classified as proficient did so.

## 7 Discussion: What is the geography of climate science for adaptation?

Our examination of climate projections reported by countries (n = 170 of 189) in their UNFCCC National Communications, between 1999 to 2016, raises fresh questions about the debates on ‘geographical imbalances’ (Pasgaard et al., 2015) and the so-called ‘north-south divide’ (Blicharska et al., 2017). In particular, our analysis characterises countries’ capacity to use and customise climate science. Our results paint a complex picture:

(a). Comparing the complexity of modelling approaches, our study supports that countries proficient in publishing climate science are also significantly more often able to use the most sophisticated method available. Dynamical downscaling requires most expertise and infrastructure, but also allows most customisation (e.g., choice of models, observation datasets, visualisation). In a study contrasting the climate projections of three leaders in climate science, Skelton et al. (2017) found that this customisation included making modelling choices influenced by the respective country’s civic epistemology and political culture in order to increase the climate projections’ national legitimacy. Furthermore, comparing preliminary and advanced nations’ climate projections reveals that the complexity of modelling approaches is only just statistically insignificant (p = .06), even though the publication competence is quite different. This is partly due to preliminary countries able to profit from pan-European modelling projects such as ENSEMBLES (van der Linden and Mitchell, 2009), and partly due to free-to-use climate model tools such as SDSM or PRECIS allowing countries with few publications to outperform nations with many publications. For instance, Bhutan and Paraguay are able to produce high-resolution climate projections with PRECIS, while Brazil and China have developed their own climate models, but reported projections using GCMs without downscaling.

(b). Our results question the so-called ‘north’ and ‘south’ binary (cf. Blicharska et al., 2017; Karlsson et al., 2007) as too simplistic to characterise countries’ capacity to customise global climate science. We found that some ‘southern’ countries have their own climate models and used a more complex modelling technique in their climate projections (e.g., Brazil, China, India, or Russia) while other ‘northern’ countries (e.g., Bahrain, Barbados, and the United Arab Emirates) failed to report climate projections altogether. The ‘north-south divide’ calls for a geographically fairer distribution in the production of climate science, but is unable to explain differences within the ‘north’ and ‘south’. Our research questions the capacity of countries to use and translate global climate science for their local context. For example, ‘lookup’ methods require no climate science expertise, while plug-and-play methods such as MAGICC-SCENGEN already allow users to select (preconfigured) climate models, timeframes and emissions pathways. Further up the line, the use of ‘GCMs only’ requires already some expertise in working with ‘raw’ climate model output as well as significant computer storage and internet bandwidth. ‘PRECIS’ meanwhile is so sophisticated that the developers (UK Met Office) run particular workshops in order to guarantee competent use as well as to ensure feedback of how reliable PRECIS output is for countries on different continents (Mahony and Hulme, 2012). With this breadth of ‘lookup’ to highly complex modelling approaches, requiring no skill to much expertise and technoscientific infrastructure, allowing no to high customisation, countries’ climate projections allow an empirically rich comparison of countries’ capacity to customise global climate science and produce nationally relevant information regardless of their peer-reviewed publication output. Such insights call for debates on ‘geographical divides’ to be extended to the uptake of climate science and its translation into national decision-support products, rather than only the origin of peer-reviewed climate science.

(c). Our results indicate – surprising given the geographical imbalance – a strong commitment by nations around the world to identify and assess climate risks with climate models, even to the point that countries with preliminary publication competence reported climate projections more often (and in higher numbers) than proficient nations (Fig. 1). Factors that have influenced such countries’ capacity to perform the scientifically more demanding parts of the National Communications include: science and technology transfer in the form of free-to-use climate modelling software such as PRECIS; free training sessions provided by UNFCCC to give expert guidance on how to prepare climate projections for the National Communications (UNFCCC, 2012, 2016); financial support to help fund the National Communication process; and countries’ requirements to ‘develop high quality [Green Climate Fund] proposals that demonstrate need [vulnerabilities]’ to increase access to financial aid tied to adaptation and mitigation (Fonta et al., 2018: 1215).

(d). The preference of the UNFCCC for climate projections to be included in National Communications (UNFCCC, 2008) may, unwittingly, introduce new geographical imbalances. Modelling initiatives such as PRECIS have been undertaken to assess climate risks in regions where little data, or scientific infrastructures, exist (Mahony and Hulme, 2012). However, making available climate science doesn’t address longer-term capacity concerns such as who becomes an IPCC author (Corbera et al., 2015; Ho-Lem et al., 2011) or who publishes in high-impact journals (Haunschild et al., 2016; Pasgaard et al., 2015). Using the example of early-career climate scientists in Africa, Dike et al. (2018) emphasise the need to improve and support internal structures for producing climate science within individual countries, for example in universities (Nasir et al., 2018). Otherwise aims such as informing adaptation policies through climate science while simultaneously basing those decisions on fairer and more locally produced scientific knowledge base remains problematic, with geopolitical implications. Although our research shows that most countries – independent of their climate science competence – seem unconcerned about using multiple climate models originating from other countries (cf. Miguel, 2017; Mahony and Hulme, 2016), they might be concerned with a lack of local customisation of such globally uniform datasets, including taking over the tool producers’ social and scientific values of ‘good’ science for decision-making (cf. Skelton et al., 2017).

Our research questions the extent to which efforts to minimise gaps in climate science *availability* may mask, or even worsen, a country’s *dependency* on climate science produced only elsewhere. For instance, the recent push towards co-produced climate services *customised* to a stakeholder’s need (cf. Vaughan and Dessai, 2014; Porter and Dessai, 2017; Bruno Soares and Buontempo, 2019; Skelton et al., 2019) is very difficult to achieve when countries rely on pre-configured climate modelling software. While in the near future the means of accessing climate science might well change, as modelling software such as MAGICC-SCENGEN is discontinued and climate information websites are evolving (Hewitson et al., 2017), customisation restrictions will likely continue.

Future research should critically examine what interrelated factors maintain, shift and potentially worsen the geographical imbalance in countries’ capacity to customise global climate science. Based on our research, five factors play an important role: (i) UNFCCC’s reporting requirements mirroring IPCC’s epistemic focus on climate models for risk assessments; (ii) ‘goodwill’ efforts by leading climate scientists in rich, high emissions countries (predominantly Anglophone); (iii) capacity-building commitments from climate science leaders within UNFCCC; (iv) UNFCCC assistance provided to non-Annex 1 countries with fewer climate science publications when preparing National Communications; and (v) countries’ improved access to financial aid (e.g. GCF) following vulnerability assessments.

## 8 Conclusion

Analysing individual countries’ capacity to use existing global climate science for informing national decision-making, our research supports a geographical imbalance. Most countries – irrespective of their climate science publication competence – are able to produce climate projections with similar modelling principles. While countries with less publication experience are now gaining valuable experience in *using* scientific climate knowledge, especially free-to-use modelling software, they haven’t as yet developed the capacity to customise globally uniform datasets. These countries, as a result, remain *dependent* on the climate models, expertise and tools to assess climate risks from scientifically leading countries, and have to tacitly accept what constitutes ‘good’ science for decision-making. Although climate modelling tools improve the availability of global climate science they may also contribute to a growing divide in the capacity of countries to customise science to their national contexts.

## Footnotes

1 In this article, we use the term ‘climate futures’ to spotlight ways of envisioning futures in order to discuss climate change today. As such, ‘climate futures’ emphasise multiple socio-economic and temporal frames used to describe climate change in the future. We introduce ‘climate futures’ to make clear we aren’t comparing plausible future states of the climate in a particular region (‘future climates’).

2 Throughout this study, we only analyse the use of knowledge derived from climate science. This has, in part, to do with other types of climate knowledge, for instance indigenous, experiential or artistic (cf. Engels, 2019), not included in our UNFCCC dataset. However, as the word’s origin highlights, *custom*isation of climate science for national adaptation could well include weaving in other nationally prevalent forms of complementary climate knowledges into climate projections.

3 Iraq’s submitted National Communication was unavailable for download. Iraq was thus not considered in the 189 submissions.

4 The Australian omission is likely associated with new climate projections being developed at the time, and not tied to political reasons. Australia’s Sixth National Communication was prepared under the outgoing Labour administration favouring climate action.
