# Supplementary material for: Customising global climate science for national adaptation: A case study of climate projections in UNFCCC’s National Communications
Source: Environ Sci Policy. 2019 Nov;101:16–23. doi: 10.1016/j.envsci.2019.07.015 (PMC6853413; doi:10.1016/j.envsci.2019.07.015)
Supplement: Supplementary file 1 [file mmc1.doc]

# Supplementary Figure for:

Skelton M, Porter JJ, Dessai S, Bresch DN, Knutti R, 2019. Customising global climate science for national adaptation: A case study of climate projections in UNFCCC’s National Communications. *Environmental Science and Policy*. DOI: 10.1016/j.envsci.2019.07.015


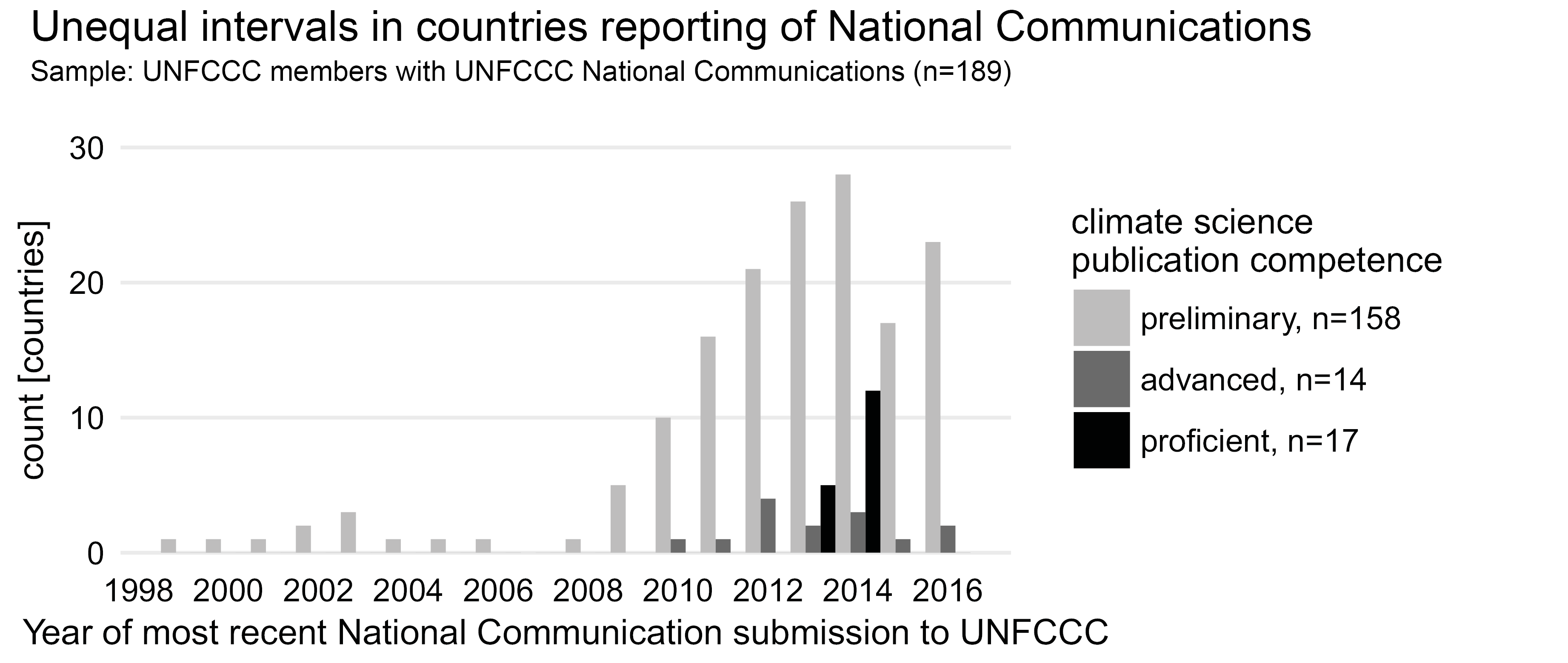


[Supplementary Figure 1 – Distribution of the submission year of UNFCCC members’ most recent National Communication (as of 31.12.2016). Note the positively skewed distribution after 2012.]
