# Supplementary material for: Customising global climate science for national adaptation: A case study of climate projections in UNFCCC’s National Communications
Source: Environ Sci Policy. 2019 Nov;101:16–23. doi: 10.1016/j.envsci.2019.07.015 (PMC6853413; doi:10.1016/j.envsci.2019.07.015)
Supplement: Supplementary file 2 [file mmc2.doc]

# Supplementary Materials for:

Skelton M, Porter JJ, Dessai S, Bresch DN, Knutti R, 2019. Customising global climate science for national adaptation: A case study of climate projections in UNFCCC’s National Communications. *Environmental Science and Policy*. DOI: 10.1016/j.envsci.2019.07.015

# List of characteristics assessed

## General country characteristics

- Country short name
- Full country name
- 3-letter country code
- Is the country a High Income country as classified by the World Bank? (For north-south definition as used in Blicharska et al. 2017)
- Is the country an OECD member? (for north-south definition as used in Blicharska et al. 2017)
- Is the country classified as northern or southern? (see Blicharska et al. 2017)
- GDP per capita 2000-2009 (Pasgaard & Strange 2013)
- UNFCCC Annex I membership / UNFCCC non-Annex I membership

## Data on most recent National Communication [until 31.12.2016]

- National Communication issue, version, and submission date
- UNDP/GEF financing of National Communication efforts

## Characteristics of climate projections

- Number of sets of climate projections reported
- Total page count for climate projections
- Method & models used for climate projection
- What type of modelling complexity was used? [noCP / other / lookup / plug-and-play / GCM only / statistical downscaling / PRECIS / dynamical downscaling]
- What underlying type of modelling characteristics was used? [model-based / GCM / RCM]
- Count of number of GCMs used
- Count of number of RCMs used
- How many timeframes were reported?
- What is the earliest year reported?
- What is the latest year reported?
- How many emission scenarios were reported?
- If only a single emission scenario was reported, which one was?

**Supplementary Table 1:** Key characteristics of countries’ climate projections, sorted first by their climate science publication competence (Haunschild et al. 2016), then alphabetically.

| Country Name | ISO3 country code | Climate Science Publication Competence | National Communication issue | Submission year of NC | Sets of Climate Projections reported | Climate Modelling Complexity | number of GCMs | number of RCMs | Number of Timeframes | Number of Emissions Pathways |
| --- | --- | --- | --- | --- | --- | --- | --- | --- | --- | --- |
| Afghanistan | AFG | preliminary | NC_1 | 2013 | 1 | 2_lookup |  |  | 3 | 1 |
| Albania | ALB | preliminary | NC_3 | 2016 | 1 | 3_plugandplay | 5 | 0 | 3 | 5 |
| Algeria | DZA | preliminary | NC_2 | 2010 | 3 | 4_GCM | 1 | 0 | 2 | 1 |
| Angola | AGO | preliminary | NC_1 | 2012 | 0 | 0_noCP |  |  |  |  |
| Antigua and Barbuda | ATG | preliminary | NC_3 | 2016 | 1 | 2_lookup |  |  | 3 | 3 |
| Armenia | ARM | preliminary | NC_3 | 2015 | 1 | 4_GCM | 1 | 0 | 3 | 2 |
| Azerbaijan | AZE | preliminary | NC_3 | 2016 | 2 | 3_plugandplay | 1 | 0 | 2 | 2 |
| Bahamas | BHS | preliminary | NC_2 | 2015 | 2 | 3_plugandplay | 21 | 0 | 1 | 1 |
| Bahrain | BHR | preliminary | NC_2 | 2012 | 0 | 0_noCP |  |  |  |  |
| Bangladesh | BGD | preliminary | NC_2 | 2012 | 1 | 3_plugandplay | 9 | 0 | 2 | 2 |
| Barbados | BRB | preliminary | NC_1 | 2001 | 0 | 0_noCP |  |  |  |  |
| Belarus | BLR | preliminary | NC_6 | 2015 | 1 | 4_GCM |  |  | 3 | 3 |
| Belize | BLZ | preliminary | NC_3 | 2016 | 2 | 6_PRECIS | 2 | 1 | 1 | 1 |
| Benin | BEN | preliminary | NC_2 | 2011 | 1 | 3_plugandplay | 4 | 0 | 4 | 2 |
| Bhutan | BTN | preliminary | NC_2 | 2011 | 1 | 6_PRECIS | 2 | 1 | 2 | 1 |
| Bolivia | BOL | preliminary | NC_2 | 2009 | 3 | 4_GCM | 1 | 0 | 2 | 1 |
| Bosnia and Herzegovina | BIH | preliminary | NC_2 | 2013 | 1 | 7_dyn.downsc | 2 | 1 | 2 | 2 |
| Botswana | BWA | preliminary | NC_2 | 2013 | 1 | 3_plugandplay | 10 | 0 | 1 | 1 |
| Brunei Darussalam | BRN | preliminary | NC_1 | 2016 | 2 | 7_dyn.downsc | 6 | 1 | 2 | 1 |
| Bulgaria | BGR | preliminary | NC_6 | 2014 | 2 | 7_dyn.downsc | 2 | 1 | 1 | 2 |
| Burkina Faso | BFA | preliminary | NC_2 | 2015 | 1 | 1_no.description |  |  | 1 | 1 |
| Burundi | BDI | preliminary | NC_2 | 2010 | 1 | 1_no.description |  | 0 | 5 | 1 |
| Cabo Verde | CPV | preliminary | NC_2 | 2011 | 1 | 1_no.description |  | 0 | 2 |  |
| Cambodia | KHM | preliminary | NC_2 | 2016 | 2 | 3_plugandplay | 2 | 0 | 3 | 2 |
| Cameroon | CMR | preliminary | NC_2 | 2016 | 1 | 2_lookup |  |  | 3 | 1 |
| Central African Republic | CAF | preliminary | NC_2 | 2015 | 0 | 0_noCP |  |  |  |  |
| Chad | TCD | preliminary | NC_2 | 2013 | 1 | 3_plugandplay | 3 | 0 | 3 | 2 |
| Chile | CHL | preliminary | NC_2 | 2011 | 1 | 6_PRECIS | 1 | 1 | 3 | 2 |
| Colombia | COL | preliminary | NC_2 | 2010 | 1 | 6_PRECIS | 5 | 3 | 2 | 3 |
| Comoros | COM | preliminary | NC_2 | 2013 | 2 | 2_lookup |  | 0 | 3 | 3 |
| Congo, Democratic Republic of the | COD | preliminary | NC_3 | 2015 | 1 | 5_stat_downsc | 3 | 0 | 2 | 2 |
| Congo, Republic of the | COG | preliminary | NC_2 | 2009 | 1 | 3_plugandplay | 5 | 0 | 6 | 2 |
| Cook Islands | COK | preliminary | NC_2 | 2012 | 1 | 3_plugandplay | 11 | 0 | 3 | 3 |
| Costa Rica | CRI | preliminary | NC_3 | 2014 | 0 | 0_noCP |  |  |  |  |
| Côte d'Ivoire | CIV | preliminary | NC_2 | 2010 | 1 | 7_dyn.downsc |  | 1 | 3 | 1 |
| Croatia | HRV | preliminary | NC_6 | 2014 | 2 | 7_dyn.downsc | 6 | 12 | 3 | 1 |
| Cuba | CUB | preliminary | NC_2 | 2015 | 1 | 6_PRECIS | 2 | 1 | 1 | 2 |
| Cyprus | CYP | preliminary | NC_6 | 2013 | 1 | 7_dyn.downsc | 5 | 7 | 2 | 3 |
| Czech Republic | CZE | preliminary | NC_6 | 2014 | 1 | 7_dyn.downsc |  | 1 | 3 | 1 |
| Djibouti | DJI | preliminary | NC_2 | 2014 | 1 | 4_GCM | 3 | 0 | 1 | 1 |
| Dominica | DMA | preliminary | NC_2 | 2012 | 1 | 7_dyn.downsc | 3 | 1 | 4 | 3 |
| Dominican Republic | DOM | preliminary | NC_2 | 2009 | 0 | 0_noCP |  |  |  |  |
| Ecuador | ECU | preliminary | NC_2 | 2012 | 1 | 7_dyn.downsc | 3 | 2 | 2 | 3 |
| Egypt | EGY | preliminary | NC_3 | 2016 | 0 | 0_noCP |  |  |  |  |
| El Salvador | SLV | preliminary | NC_2 | 2013 | 1 | 4_GCM | 6 |  | 5 | 2 |
| Eritrea | ERI | preliminary | NC_2 | 2012 | 2 | 2_lookup | 21 | 0 | 3 | 2 |
| Estonia | EST | preliminary | NC_6 | 2014 | 1 | 7_dyn.downsc | 2 | 1 |  | 2 |
| Ethiopia | ETH | preliminary | NC_2 | 2016 | 2 | 2_lookup |  |  | 3 | 3 |
| Fiji | FJI | preliminary | NC_2 | 2014 | 1 | 4_GCM | 16 |  | 3 | 1 |
| Gabon | GAB | preliminary | NC_2 | 2011 | 1 | 3_plugandplay |  | 0 | 2 |  |
| Gambia | GMB | preliminary | NC_2 | 2013 | 1 | 4_GCM | 3 | 0 | 10 | 1 |
| Georgia | GEO | preliminary | NC_3 | 2016 | 1 | 7_dyn.downsc | 1 | 1 | 2 | 1 |
| Ghana | GHA | preliminary | NC_3 | 2015 | 1 | 7_dyn.downsc | 3 | 8 | 3 | 1 |
| Grenada | GRD | preliminary | NC_1 | 2000 | 0 | 0_noCP |  |  |  |  |
| Guatemala | GTM | preliminary | NC_2 | 2016 | 1 | 4_GCM | 1 | 0 | 4 | 2 |
| Guinea | GIN | preliminary | NC_1 | 2002 | 1 | 4_GCM | 3 | 0 | 5 | 3 |
| Guinea-Bissau | GNB | preliminary | NC_2 | 2011 | 1 | 4_GCM | 5 | 0 | 2 | 2 |
| Guyana | GUY | preliminary | NC_2 | 2012 | 1 | 2_lookup | 15 |  | 3 | 3 |
| Haiti | HTI | preliminary | NC_2 | 2013 | 1 | 6_PRECIS |  | 1 | 2 | 2 |
| Honduras | HND | preliminary | NC_2 | 2012 | 2 | 4_GCM | 5 | 0 | 3 | 2 |
| Hungary | HUN | preliminary | NC_6 | 2014 | 4 | 6_PRECIS |  | 1 | 1 | 3 |
| Iceland | ISL | preliminary | NC_6 | 2014 | 1 | 7_dyn.downsc |  | 3 | 1 | 1 |
| Indonesia | IDN | preliminary | NC_2 | 2012 | 2 | 4_GCM | 15 | 0 | 3 | 2 |
| Iran | IRN | preliminary | NC_2 | 2011 | 3 | 3_plugandplay | 2 | 0 | 4 | 18 |
| Iraq | IRQ | preliminary | NC_1 | 2015 | 0 | 0_noCP |  |  |  |  |
| Ireland | IRL | preliminary | NC_6 | 2014 | 1 | 7_dyn.downsc | 9 | 3 | 1 | 2 |
| Jamaica | JAM | preliminary | NC_2 | 2011 | 3 | 6_PRECIS |  | 1 | 3 | 2 |
| Jordan | JOR | preliminary | NC_3 | 2014 | 1 | 7_dyn.downsc | 9 | 2 | 3 | 2 |
| Kazakhstan | KAZ | preliminary | NC_2 | 2014 | 1 | 4_GCM | 5 | 0 | 3 | 4 |
| Kenya | KEN | preliminary | NC_2 | 2015 | 2 | 7_dyn.downsc |  | 1 | 1 | 1 |
| Kiribati | KIR | preliminary | NC_2 | 2013 | 4 | 3_plugandplay | 21 | 0 | 4 | 3 |
| Kuwait | KWT | preliminary | NC_1 | 2012 | 2 | 4_GCM | 1 | 0 | 3 | 1 |
| Kyrgyzstan | KGZ | preliminary | NC_2 | 2009 | 1 | 3_plugandplay | 17 | 0 | 1 | 2 |
| Laos | LAO | preliminary | NC_2 | 2013 | 1 | 4_GCM | 14 | 0 | 2 | 3 |
| Latvia | LVA | preliminary | NC_6 | 2013 | 1 | 1_no.description |  |  | 1 | 2 |
| Lebanon | LBN | preliminary | NC_2 | 2011 | 1 | 7_dyn.downsc | 1 | 1 | 2 | 1 |
| Lesotho | LSO | preliminary | NC_2 | 2013 | 1 | 4_GCM | 6 | 0 | 10 | 4 |
| Liberia | LBR | preliminary | NC_1 | 2013 | 1 | 7_dyn.downsc | 2 | 10 | 4 | 1 |
| Liechtenstein | LIE | preliminary | NC_6 | 2013 | 1 | 7_dyn.downsc | 8 | 14 | 1 | 3 |
| Lithuania | LTU | preliminary | NC_6 | 2014 | 1 | 1_no.description |  |  |  | 2 |
| Luxembourg | LUX | preliminary | NC_6 | 2014 | 1 | 7_dyn.downsc | 8 | 10 | 1 | 1 |
| Macedonia | MKD | preliminary | NC_3 | 2014 | 1 | 3_plugandplay | 18 | 0 | 4 | 6 |
| Madagascar | MDG | preliminary | NC_2 | 2010 | 1 | 2_lookup |  |  | 3 | 1 |
| Malawi | MWI | preliminary | NC_2 | 2012 | 1 | 3_plugandplay | 4 | 0 | 4 |  |
| Malaysia | MYS | preliminary | NC_2 | 2011 | 2 | 6_PRECIS |  | 1 | 2 |  |
| Maldives | MDV | preliminary | NC_2 | 2016 | 1 | 7_dyn.downsc | 4 | 1 | 2 | 3 |
| Mali | MLI | preliminary | NC_2 | 2012 | 1 | 3_plugandplay | 1 | 0 |  | 2 |
| Malta | MLT | preliminary | NC_2 | 2014 | 1 | 3_plugandplay | 6 | 0 | 4 | 2 |
| Marshall Islands | MHL | preliminary | NC_2 | 2015 | 1 | 1_no.description |  |  | 2 | 2 |
| Mauritania | MRT | preliminary | NC_3 | 2014 | 1 | 3_plugandplay | 2 | 0 | 2 | 2 |
| Mauritius | MUS | preliminary | NC_2 | 2011 | 1 | 3_plugandplay | 9 | 0 | 4 | 4 |
| Micronesia | FSM | preliminary | NC_2 | 2015 | 1 | 4_GCM | 18 | 0 | 3 | 3 |
| Moldova | MDA | preliminary | NC_3 | 2014 | 1 | 4_GCM | 10 | 0 | 3 | 3 |
| Monaco | MCO | preliminary | NC_6 | 2014 | 1 | 1_no.description |  |  | 3 | 2 |
| Mongolia | MNG | preliminary | NC_2 | 2010 | 2 | 4_GCM | 1 | 0 | 3 | 3 |
| Montenegro | MNE | preliminary | NC_2 | 2015 | 1 | 7_dyn.downsc |  | 1 | 2 | 2 |
| Morocco | MAR | preliminary | NC_3 | 2016 | 1 | 1_no.description |  |  | 3 | 2 |
| Mozambique | MOZ | preliminary | NC_1 | 2006 | 1 | 4_GCM | 6 | 0 | 1 | 1 |
| Myanmar | MMR | preliminary | NC_1 | 2012 | 2 | 3_plugandplay | 4 | 0 | 3 | 2 |
| Namibia | NAM | preliminary | NC_3 | 2015 | 2 | 5_stat_downsc | 10 | 0 | 1 | 1 |
| Nauru | NRU | preliminary | NC_2 | 2015 | 1 | 4_GCM | 18 | 0 | 3 | 4 |
| Nepal | NPL | preliminary | NC_2 | 2015 | 1 | 6_PRECIS | 1 | 1 | 3 | 1 |
| Nicaragua | NIC | preliminary | NC_2 | 2011 | 4 | 6_PRECIS |  | 1 | 2 | 2 |
| Niger | NER | preliminary | NC_2 | 2009 | 3 | 5_stat_downsc | 1 | 0 | 1 | 2 |
| Nigeria | NGA | preliminary | NC_2 | 2014 | 1 | 5_stat_downsc |  |  | 2 | 2 |
| Niue | NIU | preliminary | NC_2 | 2016 | 3 | 4_GCM | 23 | 0 | 1 |  |
| North Korea | PRK | preliminary | NC_2 | 2013 | 2 | 3_plugandplay | 1 | 0 | 8 | 2 |
| Oman | OMN | preliminary | NC_1 | 2013 | 1 | 7_dyn.downsc | 1 | 0 | 2 | 1 |
| Pakistan | PAK | preliminary | NC_1 | 2003 | 1 | 3_plugandplay |  | 0 | 2 | 2 |
| Palau | PLW | preliminary | NC_1 | 2003 | 1 | 4_GCM | 1 | 0 | 2 |  |
| Panama | PAN | preliminary | NC_2 | 2012 | 1 | 4_GCM | 4 | 0 | 3 | 2 |
| Papua New Guinea | PNG | preliminary | NC_2 | 2015 | 0 | 0_noCP |  |  |  |  |
| Paraguay | PRY | preliminary | NC_2 | 2011 | 1 | 6_PRECIS | 5 | 1 | 3 | 2 |
| Peru | PER | preliminary | NC_3 | 2016 | 3 | 4_GCM | 3 |  | 1 | 2 |
| Philippines | PHL | preliminary | NC_2 | 2014 | 1 | 6_PRECIS |  | 1 | 2 | 1 |
| Qatar | QAT | preliminary | NC_1 | 2011 | 0 | 0_noCP |  |  |  |  |
| Romania | ROU | preliminary | NC_6 | 2013 | 2 | 7_dyn.downsc | 9 | 9 | 1 | 1 |
| Rwanda | RWA | preliminary | NC_2 | 2012 | 1 | 3_plugandplay | 3 | 0 | 10 | 1 |
| Saint Kitts and Nevis | KNA | preliminary | NC_2 | 2016 | 1 | 5_stat_downsc | 1 | 0 | 3 | 3 |
| Saint Lucia | LCA | preliminary | NC_2 | 2012 | 1 | 7_dyn.downsc |  |  | 3 | 1 |
| Saint Vincent and the Grenadines | VCT | preliminary | NC_2 | 2016 | 2 | 2_lookup | 15 | 0 | 3 | 3 |
| Samoa | WSM | preliminary | NC_2 | 2010 | 1 | 1_no.description |  |  | 4 |  |
| San Marino | SMR | preliminary | NC_2 | 2013 | 0 | 0_noCP |  |  |  |  |
| São Tomé… | STP | preliminary | NC_2 | 2012 | 1 | 5_stat_downsc |  | 0 | 1 | 2 |
| Saudi Arabia | SAU | preliminary | NC_2 | 2011 | 2 | 6_PRECIS | 3 | 1 | 1 | 1 |
| Senegal | SEN | preliminary | NC_3 | 2016 | 1 | 7_dyn.downsc |  | 1 | 2 | 1 |
| Serbia | SRB | preliminary | NC_1 | 2010 | 1 | 7_dyn.downsc |  | 1 | 2 | 2 |
| Seychelles | SYC | preliminary | NC_2 | 2013 | 1 | 3_plugandplay | 7 | 0 | 3 | 2 |
| Sierra Leone | SLE | preliminary | NC_2 | 2012 | 1 | 3_plugandplay | 4 | 0 | 4 | 1 |
| Singapore | SGP | preliminary | NC_3 | 2014 | 1 | 1_no.description |  |  | 1 | 1 |
| Slovakia | SVK | preliminary | NC_6 | 2014 | 1 | 7_dyn.downsc | 2 | 2 | 1 | 3 |
| Slovenia | SVN | preliminary | NC_6 | 2014 | 0 | 0_noCP |  |  |  |  |
| Solomon Islands | SLB | preliminary | NC_1 | 2004 | 1 | 4_GCM | 3 | 0 | 2 | 1 |
| Sri Lanka | LKA | preliminary | NC_2 | 2012 | 2 | 4_GCM | 3 | 0 | 2 | 3 |
| Sudan | SDN | preliminary | NC_2 | 2013 | 1 | 5_stat_downsc | 9 | 0 | 2 | 3 |
| Suriname | SUR | preliminary | NC_2 | 2016 | 1 | 2_lookup |  |  |  | 2 |
| Swaziland | SWZ | preliminary | NC_3 | 2016 | 2 | 5_stat_downsc | 7 | 0 | 2 | 1 |
| Syria | SYR | preliminary | NC_1 | 2010 | 2 | 4_GCM | 1 |  | 3 | 2 |
| Tajikistan | TJK | preliminary | NC_3 | 2014 | 1 | 4_GCM | 3 |  |  | 3 |
| Tanzania | TZA | preliminary | NC_1 | 2003 | 1 | 4_GCM | 5 | 0 |  | 1 |
| Thailand | THA | preliminary | NC_2 | 2011 | 4 | 6_PRECIS | 1 | 1 |  |  |
| Timor-Leste | TLS | preliminary | NC_1 | 2014 | 2 | 4_GCM | 20 |  | 3 | 4 |
| Togo | TGO | preliminary | NC_3 | 2015 | 1 | 3_plugandplay | #NV | 0 | 4 | 2 |
| Tonga | TON | preliminary | NC_2 | 2012 | 2 | 3_plugandplay | 1 | 0 | 3 | 2 |
| Trinidad and Tobago | TTO | preliminary | NC_2 | 2013 | 1 | 6_PRECIS |  | 1 | 3 | 2 |
| Tunisia | TUN | preliminary | NC_2 | 2014 | 1 | 4_GCM | 4 | 0 | 2 | 4 |
| Turkmenistan | TKM | preliminary | NC_3 | 2016 | 1 | 3_plugandplay | 2 | 0 | 5 | 2 |
| Tuvalu | TUV | preliminary | NC_1 | 1999 | 1 | 4_GCM | 4 | 0 | 3 | 2 |
| Uganda | UGA | preliminary | NC_2 | 2002 | 1 | 4_GCM | 20 |  | 2 | 2 |
| Ukraine | UKR | preliminary | NC_6 | 2013 | 1 | 7_dyn.downsc | 6 | 14 | 3 | 1 |
| United Arab Emirates | ARE | preliminary | NC_3 | 2013 | 0 | 0_noCP |  |  |  |  |
| Uruguay | URY | preliminary | NC_4 | 2016 | 1 | 4_GCM |  |  | 2 | 2 |
| Uzbekistan | UZB | preliminary | NC_2 | 2008 | 1 | 3_plugandplay | 6 | 0 | 3 | 4 |
| Vanuatu | VUT | preliminary | NC_2 | 2016 | 1 | 4_GCM | 18 | 0 | 3 | 3 |
| Venezuela | VEN | preliminary | NC_1 | 2005 | 1 | 3_plugandplay | 2 | 0 | 3 | 2 |
| Viet Nam | VNM | preliminary | NC_2 | 2010 | 2 | 3_plugandplay |  | 0 | 3 | 3 |
| Yemen | YEM | preliminary | NC_2 | 2013 | 1 | 4_GCM | 3 | 0 | 1 |  |
| Zambia | ZMB | preliminary | NC_2 | 2014 | 1 | 4_GCM | 3 | 0 | 1 |  |
| Zimbabwe | ZWE | preliminary | NC_2 | 2013 | 1 | 4_GCM | 1 | 0 | 1 | 2 |
|  |  |  |  |  |  |  |  |  |  |  |
| Argentina | ARG | advanced | NC_3 | 2015 | 5 | 4_GCM | 42 | 0 | 2 | 2 |
| Brazil | BRA | advanced | NC_3 | 2016 | 2 | 4_GCM | 4 | 0 | 1 | 1 |
| China | CHN | advanced | NC_2 | 2012 | 1 | 4_GCM | 11 | 0 | 2 | 2 |
| Greece | GRC | advanced | NC_6 | 2013 | 2 | 7_dyn.downsc |  | 1 | 2 | 1 |
| India | IND | advanced | NC_2 | 2012 | 1 | 6_PRECIS | 1 | 1 | 3 | 1 |
| Israel | ISR | advanced | NC_2 | 2010 | 2 | 7_dyn.downsc | 1 | 1 | 1 | 2 |
| Japan | JPN | advanced | NC_6 | 2014 | 1 | 1_no.description |  |  |  | 3 |
| Mexico | MEX | advanced | NC_5 | 2012 | 1 | 4_GCM | 1 | 0 | 1 | 1 |
| Poland | POL | advanced | NC_6 | 2014 | 1 | 7_dyn.downsc | 4 | 8 | 2 | 1 |
| Russia | RUS | advanced | NC_6 | 2014 | 0 | 0_noCP |  |  |  |  |
| South Africa | ZAF | advanced | NC_2 | 2011 | 3 | 5_stat_downsc | 9 | 0 | 2 | 1 |
| South Korea | KOR | advanced | NC_3 | 2012 | 1 | 7_dyn.downsc |  | 1 | 1 | 1 |
| Spain | ESP | advanced | NC_6 | 2013 | 0 | 0_noCP |  |  |  |  |
| Turkey | TUR | advanced | NC_6 | 2016 | 1 | 7_dyn.downsc | 3 | 1 | 3 | 3 |
|  |  |  |  |  |  |  |  |  |  |  |
| Australia | AUS | proficient | NC_6 | 2013 | 0 | 0_noCP |  |  |  |  |
| Austria | AUT | proficient | NC_6 | 2014 | 0 | 0_noCP |  |  |  |  |
| Belgium | BEL | proficient | NC_6 | 2014 | 1 | 7_dyn.downsc |  |  | 3 | 1 |
| Canada | CAN | proficient | NC_6 | 2014 | 0 | 0_noCP |  |  |  |  |
| Denmark | DNK | proficient | NC_6 | 2014 | 1 | 7_dyn.downsc |  |  | 2 | 4 |
| Finland | FIN | proficient | NC_6 | 2013 | 1 | 4_GCM | 28 | 0 | 2 | 2 |
| France | FRA | proficient | NC_6 | 2013 | 1 | 4_GCM | 2 | 0 | 3 | 2 |
| Germany | DEU | proficient | NC_6 | 2014 | 1 | 7_dyn.downsc | 8 | 12 | 2 | 1 |
| Italy | ITA | proficient | NC_6 | 2014 | 0 | 0_noCP |  |  |  |  |
| Netherlands | NLD | proficient | NC_6 | 2013 | 1 | 7_dyn.downsc | 5 | 8 | 2 | 2 |
| New Zealand | NZL | proficient | NC_6 | 2013 | 1 | 7_dyn.downsc | 12 | 1 | 2 | 1 |
| Norway | NOR | proficient | NC_6 | 2014 | 1 | 7_dyn.downsc | 2 | 2 | 2 | 2 |
| Portugal | PRT | proficient | NC_6 | 2014 | 1 | 7_dyn.downsc |  |  | 2 | 2 |
| Sweden | SWE | proficient | NC_6 | 2014 | 1 | 7_dyn.downsc | 9 | 1 | 3 | 3 |
| Switzerland | CHE | proficient | NC_6 | 2014 | 1 | 7_dyn.downsc | 8 |  | 3 | 3 |
| United Kingdom | GBR | proficient | NC_6 | 2014 | 1 | 7_dyn.downsc |  |  | 3 | 3 |
| United States of America | USA | proficient | NC_6 | 2014 | 1 | 7_dyn.downsc |  |  |  | 2 |
